# Clinical Outcomes Associated with Oral Versus Intravenous Antibiotic Therapy in Emergency Department–Discharged Patients with Community-Acquired Pneumonia

**DOI:** 10.3390/jcm14228167

**Published:** 2025-11-18

**Authors:** Mohammed Alrashed, Saleh Alyousef, Bader Alamri, Omar Yousef, Hisham AlJarallah, Abdulmajeed Alshehri, Omar A. Almohammed, Ahmed Aljabri

**Affiliations:** 1 Department of Pharmacy Practice, College of Pharmacy, King Saud bin Abdulaziz University for Health Sciences, Riyadh 11426, Saudi Arabia; alyousef032@ksau-hs.edu.sa (S.A.); alamri234@ksau-hs.edu.sa (B.A.); yousef769@ksau-hs.edu.sa (O.Y.); algarallah447@ksau-hs.edu.sa (H.A.); shehriabdul@ksau-hs.edu.sa (A.A.); 2 Medical Research Core Facility and Platforms, King Abdullah International Medical Research Center (KAIMRC), Ministry of National Guard Health Affairs, Riyadh 11426, Saudi Arabia; 3 King Abdulaziz Medical City, National Guard Health Affairs, Riyadh 11426, Saudi Arabia; 4 Department of Clinical Pharmacy, College of Pharmacy, King Saud University, Riyadh 11426, Saudi Arabia; oalmohammed@ksu.edu.sa; 5 Department of Pharmacy Practice, Faculty of Pharmacy, King Abdulaziz University, Jeddah 21589, Saudi Arabia

**Keywords:** community-acquired pneumonia, emergency department, antibiotics, parenteral therapy, oral antibiotics, revisit rate, length of stay

## Abstract

**Background:** Community-acquired pneumonia (CAP) remains a leading cause of emergency department (ED) visits, hospitalizations, and mortality worldwide. The choice between oral (PO) and intravenous (IV) antibiotic administration in the ED varies based on patient presentation and provider preference, yet the impact of this choice on clinical outcomes, including revisit rates and ED length of stay (LOS), remains unclear. This study aimed to compare PO versus IV antibiotic therapy in CAP patients discharged from the ED in terms of baseline characteristics, treatment outcomes, and healthcare utilization. **Method:** This retrospective cohort study was conducted at a tertiary care ED at the Ministry of National Guard Health Affairs in Saudi Arabia. Adult patients diagnosed treated with antibiotic for CAP and discharged from the ED between 2020–2024 were included. Patients were categorized into two groups based on antibiotic administration: POIV. The primary results were ED LOS and 30-day revisit rates. Secondary outcomes included time to first antibiotic administration, fluid administration patterns, and baseline risk factors. Data was extracted from the electronic health record and analyzed using descriptive and inferential statistics. **Results:** A total of 430 patients were included, with 162 (37.7%) receiving PO antibiotics and 268 (62.3%) receiving IV antibiotics. Baseline characteristics showed higher heart rate, respiratory rate, and temperature in the IV group, suggesting more severe presentations. The mean ED LOS was similar between groups (oral: 6.5 ± 4.9 h vs. IV: 6.4 ± 4.5 h; *p* = 0.5559). However, the 30-day ED revisit rate was significantly lower in the IV group (23.1%) compared to oral group (34.0%) (*p* = 0.0146). IV fluids were administered more frequently in the IV group (60.4% vs. 22.2%). **Conclusions:** While both PO and IV antibiotic strategies resulted in similar ED LOS, IV antibiotic use was associated with a significantly lower 30-day revisit rate. These findings support the need for risk-based treatment decisions in the ED and highlight opportunities for antibiotic stewardship to improve patient outcomes.

## 1. Introduction

Community-acquired pneumonia (CAP) remains a leading cause of hospitalization, mortality, and healthcare expenditures worldwide. The clinical presentation of CAP varies significantly, ranging from mild cases that can be managed in outpatient settings to severe cases requiring intensive care. Early diagnosis and appropriate treatment stratification are critical in improving patient outcomes and reducing the burden on healthcare systems [1]. CAP can be caused by a variety of pathogens, including bacteria, viruses, and fungi, with bacterial pathogens being the most common. These bacterial pathogens are classified into three main groups: Gram-positive, Gram-negative, and atypical agents. Gram-positive bacteria such as Streptococcus pneumoniae, Staphylococcus aureus, and group A streptococci are frequently identified in CAP cases. Gram-negative pathogens include Hemophilus influenzae, Moraxella catarrhalis, and members of the Enterobacteriaceae family, while atypical agents such as Legionella, Mycoplasma pneumoniae, and Chlamydia pneumoniae contribute to a significant proportion of cases [2]. The global incidence of CAP varies by region, season, and population characteristics, with rates ranging from 1.5 to 14 cases per 1000 person-years. In the United States, the annual incidence is estimated at 24.8 cases per 10,000 adults, with older populations being at higher risk. CAP is the eighth leading cause of death overall and ranks as the leading cause of death from infectious diseases. Mortality is especially high in patients with severe pneumonia, reaching 23% in those admitted to intensive care units (ICUs) [1]. Treatment for CAP depends on the setting and severity of the disease. CURB-65 (five- items score) and pneumonia severity index (PSI: 20-items score) are two scores that are well studied and widely utilized in practice to predict 30-day mortality and needs for ICU admission in CAP [3]. For outpatients with no significant comorbidities, monotherapy with a macrolide (e.g., erythromycin, azithromycin, or clarithromycin) or doxycycline is recommended. In patients with comorbidities such as chronic heart or lung disease, diabetes, or immunosuppression, combination therapy with a respiratory fluoroquinolone or a beta-lactam plus macrolide is preferred. Inpatients with moderate CAP should receive either fluoroquinolone monotherapy or beta-lactam and macrolide combination therapy, while those admitted to ICUs require combination therapy with a beta-lactam and either a macrolide or respiratory fluoroquinolone [4,5,6].

The choice between PO and IV antibiotic therapy remains a critical decision point in CAP management, particularly in the emergency department (ED) setting, where initial clinical assessments often determine patient disposition and treatment course. While IV therapy is traditionally favored for patients with more severe presentations, oral therapy offers advantages such as earlier discharge, reduced costs, and avoidance of IV-related complications when clinically appropriate [6]. However, real-world prescribing patterns in EDs frequently reflect variability and potential overuse of IV antibiotics, even in patients eligible for oral therapy. Understanding these trends is essential to optimize clinical decision-making, enhance antibiotic stewardship, and ensure efficient resource utilization.

Accordingly, this study aims to compare the clinical outcomes of PO vs. IV antibiotics in the management of CAP within the ED. The primary objectives are to analyze the patterns of antibiotic route selection and their relationship to patient outcomes, including emergency department length of stay (LOS) and 30-day revisit rates. Secondary objectives include exploring factors influencing the choice of route of administration, such as disease severity and patient characteristics. The study aims to provide evidence that supports optimal antibiotic route selection, limits unnecessary use of IV therapy, and reinforces antimicrobial stewardship principles in emergency care settings.

## 2. Methods

### 2.1. Study Design and Setting

This retrospective cohort study was conducted at a tertiary care ED at the Ministry of National Guard Health Affairs (MNGHA) in Saudi Arabia, which manages approximately 100,000 visits annually. The ED is part of a large academic medical center offering comprehensive inpatient and outpatient services.

During the study period, no standardized protocol existed for the management of CAP. The selection and route of antibiotic therapy were determined by the treating physician’s clinical judgment. All parenteral antibiotics required pharmacist verification before administration, and patients were observed for 15–30 min afterward in accordance with institutional policy. Upon discharge, prescribed medications were dispensed to patients through the hospital’s outpatient pharmacy services.

### 2.2. Patient Selection

The inclusion criteria for this study included adult patients admitted to the ED, between 2020–2024, for the management of CAP with either oral or IV antibiotics and were discharged directly from the ED. The ICD-10 codes (J18.0–J18.9) were used through the electronic health record (EHR) to identify relevant patients. Patients were excluded if they had an ED visit for CAP in the preceding 30 days, were transferred from another healthcare facility, had incomplete or missing medical records, or had a confirmed or suspected diagnosis of COVID-19 pneumonia. To ensure a representative sample, patients were randomly selected until the target sample size was reached or all eligible cases were included.

### 2.3. Study Variables and Data Collection

Patients were stratified into two main treatment groups: those who received PO antibiotics only and those who received IV antibiotics, with or without oral therapy, during their ED visit. The primary outcomes were ED LOS and 30-day revisit rates following discharge. Secondary analyses examined factors influencing route selection, including disease severity indicators and patient characteristics. Data were obtained from the electronic health record (EHR) and included demographics, comorbidities, and clinical presentation variables such as temperature, respiratory rate, oxygen saturation. Medication administration records and discharge prescriptions were reviewed to confirm antibiotic route and regimen. In addition, manual chart reviews were conducted to verify radiologic findings, diagnostic workup, and follow-up encounters within the hospital network. ED LOS was defined as the time from patient registration to ED discharge.

### 2.4. Data Analysis

We hypothesized that the administration of IV antibiotics would be associated with a longer ED LOS without a significant reduction in revisit rates compared to PO therapy. The sample size was estimated based on prior ED data suggesting an expected LOS difference of approximately 45–60 min between treatment groups. Continuous variables, such as LOS, were analyzed using independent t-tests or analysis of variance (ANOVA) when comparing more than two groups. Categorical variables, including revisit rates, were analyzed using the chi-square test. Cox proportional hazards regression was employed to estimate hazard ratios (HRs) for ED revisits at 72 h and 30 days. Covariates included age, sex, comorbidities (e.g., COPD, diabetes), baseline vital signs, radiographic findings, and adherence to guideline-recommended therapy. A two-sided *p*-value of <0.05 was considered statistically significant. All analyses were performed using SPSS Statistics version 28 (IBM Corp., Armonk, NY, USA) [7].

## 3. Results

### 3.1. Characteristics of Study Subjects

**Figure 1:**
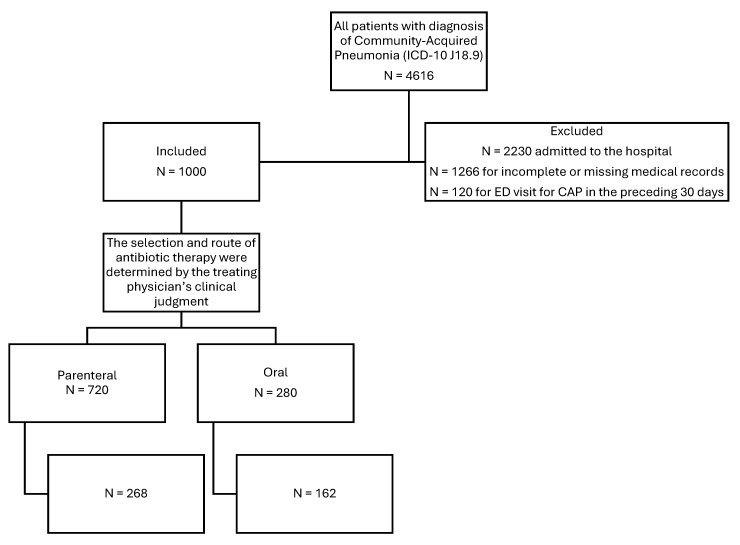
Flow diagram for study participants. (Note: Subjects were included using random number assignment until all eligible patients within the study period were enrolled).

A total of 430 adult patients diagnosed with CAP and discharged from the ED were included in the study. Patients were categorized based on the route of antibiotic administration received in the ED: 162 patients (37.7%) received PO antibiotics, and 268 patients (62.3%) received IV antibiotics (see Figure 1). Summary demographic, clinical, and laboratory characteristics are presented in Table 1. The median age of the study population was 66 years (IQR 52–76), and 51.9% were female. No statistically significant differences were found in baseline characteristics such as age, gender, BMI, or most comorbidities between the two groups. However, patients in the IV group presented with significantly higher heart rates (96 vs. 87 bpm, *p* = 0.0002), respiratory rates (21 vs. 20 breaths/min, *p* = 0.0010), and temperatures (37.6 °C vs. 37.0 °C, *p* < 0.0001), indicating more severe clinical presentation. Common presenting complaints included cough (81.4%) and fever (66.0%), with significantly more patients in the IV group presenting with these symptoms (*p* = 0.0010 and *p* < 0.0001, respectively). Risk factors for recurrence, such as age ≥65, diabetes, hypertension, and obesity, were similarly distributed between groups.

### 3.2. Main Results

**Figure 2:**
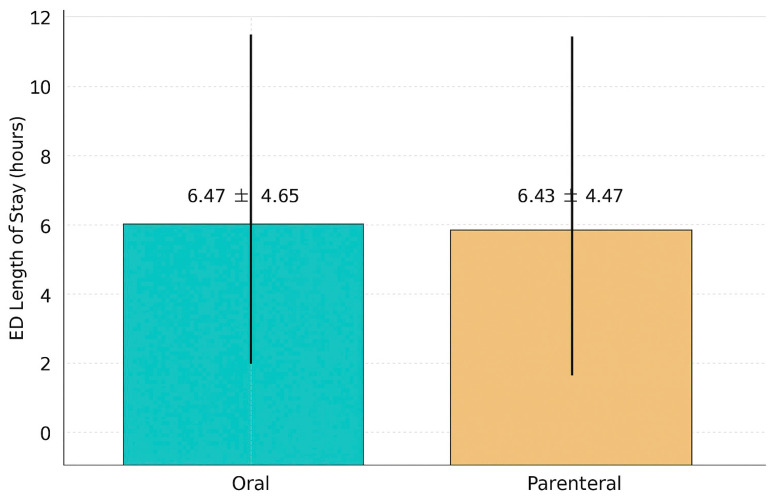
Mean Emergency Department LOS for Patients with Community-Acquired Pneumonia.

**Figure 3:**
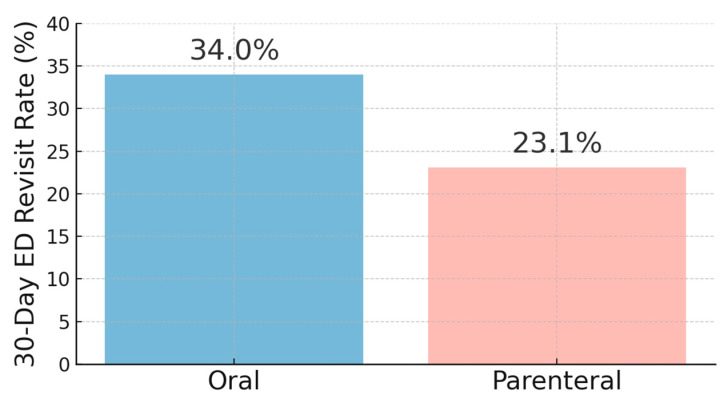
30-Day Emergency Department Revisit Rates for Patients Receiving Oral vs. Parenteral Therapy.

Medical interventions and outcomes are summarized in Table 2 and Table 3. The mean ED LOS was similar between the groups: 6 h and 31 min (±4:56) in the PO group and 6 h and 26 min (±4:28) in the IV group (*p* = 0.5559), indicating that parenteral therapy did not significantly prolong ED stay (Figure 2). Parenteral antibiotics were administered promptly, with an average time to initiation of 13:39 ± 6:52 h from ED arrival. Common IV agents included ceftriaxone alone (23.7%) or in combination with azithromycin (21.9%), while oral regimens often included moxifloxacin (28.6%), cefuroxime plus azithromycin (19.8%), or Augmentin-based combinations. Intravenous fluid resuscitation (0.9% NaCl) was administered in 46.0% of the overall cohort. Significantly more patients in the parenteral group received fluids compared to the oral group (60.4% vs. 22.2%). Time to fluid initiation was similar (13:00 ± 6:54 in IV group vs. 12:28 ± 6:58 in oral group). A total of 117 patients (27.2%) returned to the ED within 30 days. The revisit rate was significantly lower in the parenteral group compared to the oral group (23.1% vs. 34.0%, *p* = 0.0146). The average time to revisit did not differ significantly between groups (33.1 ± 48.4 days vs. 27.4 ± 45.8 days, *p* = 0.1578). These findings suggest that although both oral and parenteral antibiotic regimens were associated with comparable ED length of stay, patients treated with IV antibiotics had lower ED revisit rates within 30 days. The increased revisit rate in the oral group may reflect differences in illness severity or antibiotic selection, supporting the need for further evaluation of antibiotic stewardship and risk-based treatment selection in the ED setting (Figure 3).

## 4. Discussion

This study was designed to evaluate clinical outcomes associated with the route of antibiotic administration among patients presenting to the ED with CAP who were discharged home. The most important finding was that while ED LOS did not differ significantly between patients treated with IV versus PO antibiotics, the 30-day revisit rate was significantly lower in the parenteral group. These findings underscore the importance of individualized, risk-based antibiotic selection and may have implications for antimicrobial stewardship and healthcare resource utilization in the ED setting.

Unlike prior studies that focused primarily on inpatient populations or patients with more severe pneumonia [8,9,10], our cohort specifically involved ED-discharged patients—representing a less severe, yet clinically relevant, CAP subgroup. The lack of significant difference in LOS (~6.5 h in both groups) contrasts with findings in other infectious diseases such as urinary tract or soft tissue infections, where IV therapy has been associated with longer ED stays [11,12,13,14]. In our setting, most IV medications were administered as IV infusions rather than IV push, which differs from other studies where IV push was standard; nonetheless, short observation protocols likely mitigated delays. In busy EDs, even small changes in LOS can influence patient flow and operational efficiency [15].

The significantly lower 30-day revisit rate in the IV group (23.1% vs. 34.0%, *p* = 0.0146) may be attributed to several factors. Patients who received IV therapy tended to present with higher initial severity scores and were thus more likely to receive immediate, broad-spectrum of antibiotics and closer short-term monitoring before discharge. Such management may have ensured more complete early bacterial suppression and reduced short-term treatment failure. Conversely, oral regimens—although guideline-concordant—are influenced by patient adherence, variable absorption, and local resistance trends, which may contribute to higher revisit rates [8,16]. These findings are consistent with prior literature supporting the use of oral antibiotics in clinically stable CAP patients, particularly those with low CURB-65 or PSI scores [9,17,18,19,20]. For example, Rae et al. found no difference in 30-day mortality or ICU admissions between oral and IV clarithromycin, and a meta-analysis by Teng et al. reported similar clinical success rates and adverse events between oral and IV therapies, with oral regimens even associated with lower all-cause mortality [17,18].

In real-world ED practice, formal severity scoring (e.g., CURB-65, PSI) is often underused, and treatment decisions depend largely on clinical judgment. Lung ultrasound has emerged in recent years a useful tool for diagnosing and monitoring of CAP. In fact, evidence have shown that lung ultrasound has a sensitivity between 85–97% and specificity between 80–96% diagnosis for diagnosing CAP [21]. A pragmatic approach—administering a single parenteral dose before discharge with an oral step-down regimen—may represent an effective compromise for moderate-risk patients, balancing early bacterial clearance with outpatient management efficiency [21,22,23]. Such strategies align with antimicrobial stewardship principles by targeting therapy intensity to patient risk. Importantly, very few studies to date have focused exclusively on CAP management in the ED among patients discharged directly home, making this study a valuable addition to the limited evidence base on outpatient CAP treated in the ED.

### Limitations

This study has several important limitations. First, its retrospective design limits the ability to control unmeasured confounders and relies on the accuracy and completeness of documentation within the electronic health record. Variability in clinical decision-making, provider experience, and documentation practices may have influenced both the choice of antibiotic route and the recorded clinical parameters. Second, although baseline characteristics were compared, residual differences in illness severity between the oral and parenteral groups may have affected outcomes such as revisit rates, as patients with more severe presentations were more likely to receive intravenous therapy. Third, antibiotic appropriateness was evaluated based on available documentation and may not fully capture adherence to institutional or international guideline recommendations. Fourth, the study period overlapped with the COVID-19 pandemic; despite excluding confirmed COVID-19 pneumonia, inadvertent inclusion or misclassification of viral or mixed bacterial–viral infections cannot be completely excluded. Fifth, because follow-up data outside the MNGHA hospital network was unavailable, revisit rates may have been underestimated. Lastly, the single-center design and lack of comprehensive microbiologic data limit the generalizability of the findings and preclude pathogen-specific outcome assessment.

These limitations highlight the need for multicenter, prospective studies to confirm these associations and further define optimal antibiotic selection strategies for CAP patients discharged from the emergency department.

## 5. Conclusions

This retrospective study highlights that while oral and parenteral antibiotics for community-acquired pneumonia in the emergency department resulted in similar lengths of stay, patients receiving parenteral therapy had significantly lower 30-day ED revisit rates. These findings suggest that the route of antibiotic administration may be associated with differences in clinical outcomes and underscore the importance of risk-based decision-making in antibiotic selection. Although parenteral antibiotics were more frequently used in patients with more severe presentations, the observed lower revisit rate may reflect the influence of initial management practices rather than a direct treatment effect. The results emphasize the need for integrating clinical severity assessment tools and guideline-concordant prescribing into ED practice to optimize patient care and resource utilization. Further prospective, multicenter studies are warranted to validate these associations and inform evidence-based antibiotic stewardship strategies.

## Figures and Tables

**Table 1:** Baseline Characteristics for patients with community acquired pneumonia (CAP).

Characteristics	Overall	Oral *n* = 162 (37.7)	Parenteral *n* = 268 (62.3)	*p*-Value
Age, years	66.0 (52.0–76.0)	67.0 (54.0–77.0)	65.0 (51.0–75.0)	0.3587
Gender, Female	223 (51.9)	77 (47.5)	146 (54.5)	0.1624
Weight, kg	77.0 (66.0–92.1)	76.0 (65.0–91.0)	78.0 (68.0–94.0)	0.2890
Height, cm	160.0 (153.0–166.0)	160.0 (153.0–168.0)	160.0 (153.0–166.0)	0.6676
BMI, kg/m^2^	30.3 (25.9–36.0)	30.0 (25.6–35.0)	30.8 (26.0–36.1)	0.2926
Vital signs and laboratory values				
SBP	130.0 (117.0–143.0)	133.0 (120.0–146.0)	128.5 (116.0–141.5)	0.0341
DBP	67.0 (59.0–76.0)	66.0 (58.0–76.0)	67.0 (60.0–77.0)	0.2686
HR	94.0 (81.0–106.0)	87.0 (78.0–100.0)	96.0 (85.0–109.5)	0.0002
RR	21.0 (20.0–23.0)	20.0 (20.0–22.0)	21.0 (20.0–24.0)	0.0010
O_2_ Saturation, %	96.0 (95.0–98.0)	96.0 (95.0–98.0)	96.0 (94.0–98.0)	0.0655
Temperature, °C	37.1 (36.8–38.1)	37.0 (36.7–37.4)	37.6 (37.0–38.6)	<0.0001
WBC	8.2 (6.0–11.4)	8.2 (6.3–10.7)	8.2 (5.7–11.9)	0.6319
CrCl	69.0 (49.6–96.0)	66.9 (43.9–94.2)	72.0 (51.5–96.5)	0.0825
eGFR	91.0 (69.0–109.0)	86.0 (60.0–105.0)	93.5 (71.0–109.0)	0.0604
BUN	4.7 (3.4–6.0)	5.0 (3.6–6.6)	4.5 (3.2–5.8)	0.0074
Presenting complaint				
Cough	350 (81.4)	119 (73.5)	231 (86.2)	0.0010
Fever	284 (66.0)	83 (51.2)	201 (75.0)	<0.0001
Shortness of breath	271 (63.0)	94 (58.0)	177 (66.0)	0.0950
Mucus production	135 (31.4)	48 (29.6)	87 (32.5)	0.5396
Chest pain	49 (11.4)	20 (12.3)	29 (10.8)	0.6297
Confusion	6 (1.4)	3 (1.9)	3 (1.1)	0.5304
Other	116 (27.0)	39 (24.1)	77 (28.7)	-
Risk factor for recurrence				
Age > or equal to 65	226 (52.6)	89 (54.9)	137 (51.1)	0.4422
Hypertension	226 (52.6)	94 (58.0)	132 (49.3)	0.0776
Diabetes	225 (52.3)	89 (54.9)	136 (50.7)	0.3990
Obesity	206 (47.9)	70 (43.2)	136 (50.7)	0.1295
Dyslipidemia	126 (29.3)	51 (31.5)	75 (28.0)	0.4402
CAD	72 (16.7)	31 (19.1)	41 (15.3)	0.3017
Asthma	55 (12.8)	17 (10.5)	38 (14.2)	0.2675
Renal diseases	45 (10.5)	23 (14.2)	22 (8.2)	0.0493
Received paren. antibiotics within 90 days	46 (10.7)	23 (14.2)	23 (8.6)	0.0679
Atrial fibrillation	40 (9.3)	16 (9.9)	24 (9.0)	0.7499
COPD	14 (3.3)	4 (2.5)	10 (3.7)	0.4748
Dementia	9 (2.1)	3 (1.9)	6 (2.2)	0.7859
Stroke	5 (1.2)	1 (0.6)	4 (1.5)	0.4120
GERD	5 (1.2)	1 (0.6)	4 (1.5)	0.4120

Data are presented as median (Interquartile range) for continuous variables or frequency (percentage) for categorical variables. CAD: Coronary artery disease; COPD: chronic obstructive pulmonary disease; BMI: body mass index; BUN: blood urea nitrogen; CrCl: creatinine clearance; DBP: diastolic blood pressure; eGFR: estimated glomerular filtration rate; GERD: gastroesophageal reflux disease; HR: heart rate; O_2_: oxygen; RR: respiratory rate; SBP: systolic blood pressure; WBC: white blood count.

**Table 2:** Medical intervention for patients with community acquired pneumonia (CAP).

Intervention	Overall	Oral *n* = 162 (37.7)	Parenteral *n* = 268 (62.3)
Emergency department resuscitation (NaCl 0.9%)	198 (46.0)	36 (22.2)	162 (60.4)
Time to start fluid resuscitation, HH:MM	12:54 ± 6:54	12:28 ± 6:58	13:00 ± 6:54
Time to start parenteral antibiotic in ED, HH:MM	13:39 ± 6:52	NA	13:39 ± 6:52
Parenteral antibiotic administered in ED			
Ceftriaxone	102 (23.7)	NA	102 (38.1)
Ceftriaxone + Azithromycin	94 (21.9)	NA	94 (35.1)
Moxifloxacin	33 (7.7)	NA	33 (12.3)
Azithromycin	16 (3.7)	NA	16 (6.0)
Piperacillin/tazobactam	11 (2.6)	NA	11 (4.1)
Piperacillin/tazobactam + Vancomycin	3 (0.7)	NA	3 (1.1)
Ceftriaxone + Clindamycin	1 (0.2)	NA	1 (0.4)
Ceftriaxone + Erythromycin	1 (0.2)	NA	1 (0.4)
Ceftriaxone + Moxifloxacin	1 (0.2)	NA	1 (0.4)
Moxifloxacin + Vancomycin	1 (0.2)	NA	1 (0.4)
Cefepime	1 (0.2)	NA	1 (0.4)
Ciprofloxacin	1 (0.2)	NA	1 (0.4)
Meropenem	1 (0.2)	NA	1 (0.4)
Ampicillin/sulbactam	1 (0.2)	NA	1 (0.4)
Prescribed oral antibiotics at discharge from ED			
Moxifloxacin	123 (28.6)	53 (32.7)	70 (26.1)
Cefuroxime + Azithromycin	85 (19.8)	24 (14.8)	61 (22.8)
Amoxicillin/clavulanic acid + Azithromycin	70 (16.3)	12 (7.4)	58 (21.6)
Amoxicillin/clavulanic acid	62 (14.4)	28 (17.3)	34 (12.7)
Cefuroxime	33 (7.7)	18 (11.1)	15 (5.6)
Azithromycin	29 (6.7)	17 (10.5)	12 (4.5)
Amoxicillin/clavulanic acid + Doxycycline	2 (0.5)	0 (0.0)	2 (0.7)
Amoxicillin/clavulanic acid + Moxifloxacin	2 (0.5)	1 (0.6)	1 (0.4)
Azithromycin + Amoxicillin	1 (0.2)	0 (0.0)	1 (0.4)
Azithromycin + Moxifloxacin	3 (0.7)	0 (0.0)	3 (1.1)
Azithromycin + Clindamycin	1 (0.2)	0 (0.0)	1 (0.4)
Cefuroxime + Doxycycline	6 (1.4)	2 (1.2)	4 (1.5)
Cefuroxime + Clarithromycin	2 (0.5)	0 (0.0)	2 (0.7)
Cefuroxime + Augmentin	1 (0.2)	0 (0.0)	1 (0.4)
Moxifloxacin + Metronidazole	2 (0.5)	2 (1.2)	0 (0.0)
Ciprofloxacin	3 (0.7)	1 (0.6)	2 (0.7)
Doxycycline	3 (0.7)	3 (1.9)	0 (0.0)
Amoxicillin	1 (0.2)	0 (0.0)	1 (0.4)
Cefprozil	1 (0.2)	1 (0.6)	0 (0.0)

Data are presented as mean ± Standard deviation for continuous variables or frequency (percentage) for categorical variables. ED: emergency department; HH:MM: hour:minute.

**Table 3:** Outcomes for patients with community acquired pneumonia (CAP).

Outcome	Overall	Oral *n* = 162 (37.7)	Parenteral *n* = 268 (62.3)	*p*-Value
Mean ED LOS, HH:MM	6:28 ± 4:39	6:31 ± 4:56	6:26 ± 4:28	0.5559
30-Day ED Revisit Rates	117 (27.2)	55 (34.0)	62 (23.1)	0.0146

Data are presented as mean ± Standard deviation for continuous variables or frequency (percentage) for categorical variables. ED: emergency department; HH:MM: hour:minute; LOS: length of stay.

## Data Availability

The datasets analyzed during the current study are available from the corresponding author upon reasonable request, subject to approval by the Institutional Review Board of King Saud bin Abdulaziz University for Health Sciences.

## Abbreviations

CAP	Community-Acquired Pneumonia
ED	Emergency Department
IV	Intravenous
PO	oral administration
LOS	Length of Stay
CURB-65	Confusion, Urea, Respiratory Rate, Blood Pressure, Age ≥65
PSI	Pneumonia Severity Index
ICU	Intensive Care Unit
COPD	Chronic Obstructive Pulmonary Disease
BMI	Body Mass Index
SBP	Systolic Blood Pressure
DBP	Diastolic Blood Pressure
HR	Heart Rate
RR	Respiratory Rate
BUN	Blood Urea Nitrogen
CrCl	Creatinine Clearance
eGFR	Estimated Glomerular Filtration Rate
WBC	White Blood Cells
HR	Hazard Ratio (in statistics section)
CV	Coefficient of Variation
OR	Odds Ratio
CI	Confidence Interval
RCTs	Randomized Controlled Trials
SPSS	Statistical Package for the Social Sciences
KAMC	King Abdulaziz Medical City
MNGHA	Ministry of National Guard Health Affairs
KAIMRC	King Abdullah International Medical Research Center
